# Single-Item and Associative Working Memory in Stroke Patients

**DOI:** 10.3233/BEN-2012-129010

**Published:** 2013

**Authors:** Bonnie van Geldorp, Roy P. C. Kessels, Marc P. H. Hendriks

**Affiliations:** ^1^Donders Institute for BrainCognition and BehaviourRadboud University NijmegenNijmegenThe Netherlands; ^2^Department of Medical PsychologyRadboud University Nijmegen Medical CentreNijmegenThe Netherlands; ^3^Department of Behavioural SciencesEpilepsy Centre KempenhaegheHeezeThe Netherlands

**Keywords:** Binding, relational memory, stroke, working memory

## Abstract

In this study, we examined working memory performance of stroke patients. A previous study assessing amnesia patients found deficits on an associative working memory task, although standard neuropsychological working memory tests did not detect any deficits. We now examine whether this may be the case for stoke patients as well. The current task contained three conditions: one spatial condition, one object condition and one binding condition in which both object and location had to be remembered. In addition, subsequent long-term memory was assessed. The results indicate that our sample of stroke patients shows a working memory deficit, but only on the single-feature conditions. The binding condition was more difficult than both single-feature conditions, but patients performed equally well as compared to matched healthy controls. No deficits were found on the subsequent long-term memory task. These results suggest that associative working memory may be mediated by structures of the medial temporal lobe.

## 1. Introduction

Stroke may affect any area of the brain resulting in a variety of cognitive deficits [1]. Identifying the profile of cognitive deficits is relevant for rehabilitation purposes for individual patients. In clinical practice, standard neuropsychological tests are used to determine working-memory deficits in patients. Typically, span-like tasks such as digit span or Corsi block tapping task are used. However, these working memory tests do not involve an associative (or binding) component. This is relevant because deficits on an associative working-memory task were found in amnesia patients who showed no deficits on the WAIS-III digit span [2]. Here, we investigate whether an associative working memory task is sensitive in stroke patients as well. Also, a subsequent memory task was administered to investigate the role of long-term encoding in relation to working memory.

## 2. Methods

### 2.1. Participants

In the present study, 24 stroke patients (mean age: 52.08, SD = 11.15, mode education level: 5, range 2–7, 18 males) and 31 matched controls (mean age: 50.65, SD = 13.99, mode education level: 5, range 2–7, 15 males) were included. All patients were recruited from the rehabilitation center Groot Klimmendaal in Arnhem, the Netherlands. The patients had all experienced an ischaemic or hemorrhagic stroke (10 left-sided, 12 right-sided and 2 bilateral). All participants provided written informed consent. The groups were matched with respect to age, gender distribution and education (all $p > 0.10$). Working memory performance, as measured with the WAIS-III digit span, did not significantly differ between patients (mean performance: 13.67, SD = 3.10) and controls (mean: 13.72, SD = 3.92; $F < 1$).

ISSN 0953-4180/13/$27.50 © 2013 – IOS Press and the authors. All rights reserved

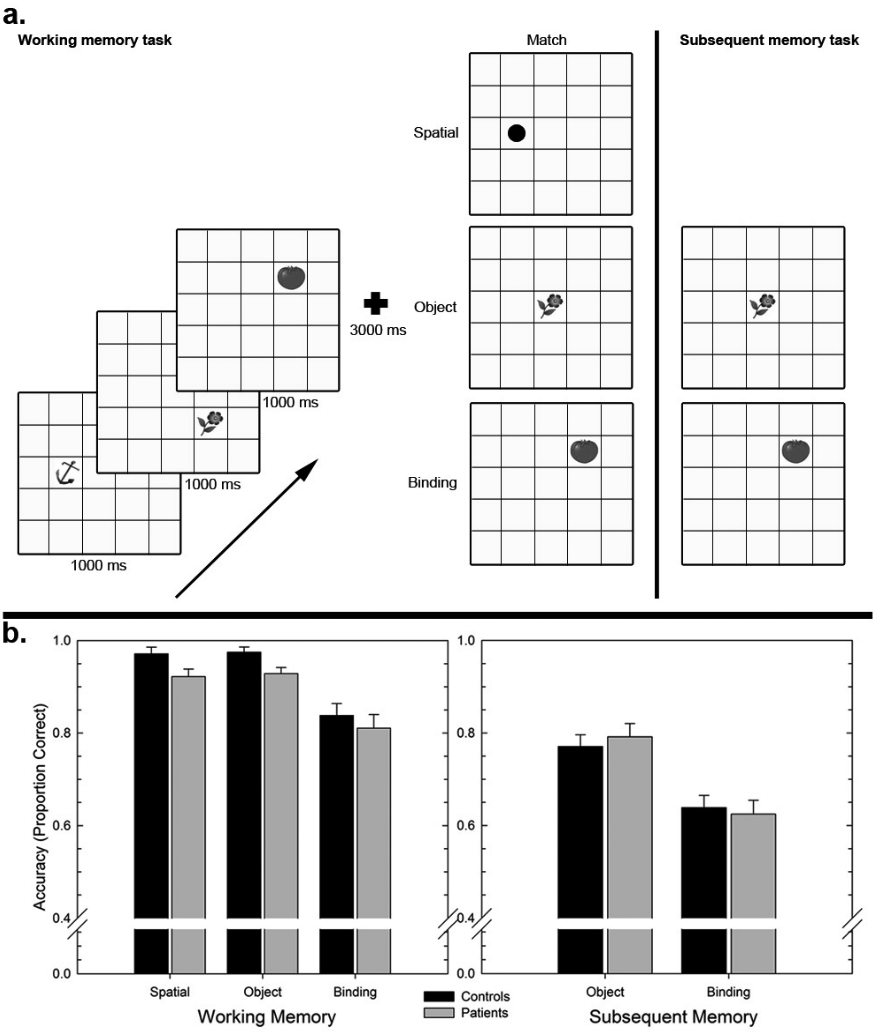

Fig. 1. a. Schematic overview of the experiment. Three stimuli are presented for 1000 ms each. After a delay period of 3000 ms, a probe is presented. Here, matching probes are shown for the three different conditions (spatial, object and binding). For the binding condition, a non-matching probe is a recombination of an object and a location presented in that trial. In the subsequent memory task, long-term memory for objects and bindings is assessed. b. Results for the working memory task and the subsequent memory task.

### 2.2. *Experimental task*

All participants completed a computerized delayed-match-to-sample task for working memory (see Fig. 1a) [3], with three blocks of 18 trials each, resulting in a total of 54 trials. In each trial, three stimuli are presented at different locations within a 5-by-5-grid for 1000 ms each. All locations on the grid are used, except for the centre location. A delay period of 3 seconds follows, in which the participant has to remember the object, location or both.

In the case of the object condition, the probe is presented as an object in the centre of the grid. The participant then has to decide whether or not it is a match to the stimuli presented in that trial. In the spatial condition, the probe is a black dot at a specific location and in the case of the binding condition it is an object at a specific location. In the latter case, the non-matching probe is a combination of a stimulus and a location from that trial, in order to minimize familiarity-based responses.

Approximately five minutes after completing this working memory task, a subsequent memory task was performed, consisting of two blocks of 12 trials each, to assess episodic memory for both objects and object-location associations. As all locations were used multiple times, we could not assess episodic memory for locations.

## 3. Results

The results are displayed in figure 1b. All participants performed significantly above chance level (all $p < 0.01$). For the working memory task, a repeated measures ANOVA with condition (object, spatial and binding) as within-subject factor and group (patient or control) as between-subject factor was performed. Since Mauchly's test showed that the assumption of sphericity was violated, we corrected the degrees of freedom, using the Greenhouse-Geisser correction.

The results show a significant effect of task condition ($F(1.59, 84.56) = 36.37, p < 0.001$), with performance on the binding condition being worse than on both the object condition ($p < 0.001$) and spatial condition ($p < 0.001$). No differences were found between the object and spatial condition. In addition, a significant group effect was observed ($F$ (1,53) $= 4.38$, $p = 0.04$). This effect seems to be largely caused by a group difference in both the spatial ($p = 0.05$) and object condition ($p = 0.02$). No group difference was observed in the binding condition ($p = 0.48$). There was no significant interaction effect of group by condition ($F < 1$).

The results of the subsequent memory task also show a significant effect of condition ($F$ (1,53) $= 29.27$, $p < 0.001$), but no significant group or interaction effect ($F < 1$). When comparing the working memory task with the subsequent memory task, we observed a significant difference between tasks ($F$ (1,53) $= 172.34$, $p < 0.001$), but no significant interaction effect of group by task ($F$(1,53) $= 2.12$, $p = 0.15$). This means that the memory performance decline that can be observed in the subsequent memory task is equal for both groups.

## 4. Discussion

The present study clearly indicates that although our sample of stroke patients in the chronic stage did not show working-memory deficits on a standard neuropsychological test, they clearly had deficits on an experimental working-memory task. However, it is interesting that the impairment lies in the single-feature conditions and not in the associative condition. These results extend previous findings showing that associative working memory may be mediated by structures of the medial temporal lobe [4]. This notion is further supported by our results showing that the groups performed equally on the subsequent long-term memory task, which relies on medial temporal-lobe function [4]. On the other hand, memory for single features may predominantly be subserved by the fronto-parietal working-memory network [5], a brain region that is more susceptible to stroke than the medial temporal lobe.

It could be argued that the associative condition has a higher memory load and may thus be more challenging than the single-feature conditions, but this appears to be equally the case for patients and controls. Also, no binding deficit was found in the patients, in contrast to the single-feature conditions. In a future study, the working memory load could be systematically varied to investigate the role of memory load. In addition, it was not possible to relate the lesion location to the pattern of impairment in our sample, but future studies should investigate the neuroanatomical substrate of single-feature vs. associative working memory in more detail.

## Acknowledgments

We thank Maaike Gaastra en Maartje Hutten for their assistance in collecting the data.

Funding: this study was funded by a VIDI innovational grant from the Netherlands Organisation for Scientific Research (NWO, no. 452-008-005), awarded to RPCK. The funders had no role in study design, data collection and analysis, decision to publish, or preparation of the manuscript.
